# Fragmentation of healthcare systems: challenges through patients’ eyes

**DOI:** 10.1080/17482631.2025.2598719

**Published:** 2025-12-16

**Authors:** Dunia Ghannam, Nathalie Angelé – Halgand, Michèle Kosremelli-Asmar

**Affiliations:** a Lebanese University, Beirut, Lebanon; b Sherbrooke University, Sherbooke, Canada; c Saint-Joseph University, Beirut, Lebanon

**Keywords:** Reification, healthcare system, expert patient, disability, care path, privatization

## Abstract

**Introduction:**

This article aims to identify key challenges raised by fragmented healthcare systems by adopting the lens of patients. We analyzed how high fragmentation leads to the loss of humanity from healthcare providers as perceived by the parents of children born with disabilities.

**Methods:**

Twenty-nine Lebanese families of children born with disability agreed to participate. Data was collected through semi-structured interviews, recorded and then coded using Nvivo 11. The coding followed an abductive approach.

**Results and contribution:**

The study revealed the financial and bureaucratic burdens, the reification and relational challenges of fragmented healthcare systems. We identified how parents became an expert patient and a care path manager. Our contribution is by addressing the fragmentation issue from the parents’ point of view. In this way, we further contribute to documenting the impact of fragmentation, on the lives of patients and their families and the intensity of the violence generated by the institutional system that amplifies the trauma inherent to the clinical case which refers to the chronic emotional and psychological distress that parents endure as they repeatedly confront care discontinuities, administrative barriers, and the need to compensate for the system’s failures.

## 1. Introduction

Healthcare systems worldwide face increasing fragmentation, characterised by a lack of coordination among providers, payers, and organisations. This systemic fragmentation often leads to inefficient resource use, suboptimal care quality, avoidable medical errors, and, in some cases, patient harm or fatalities (Cheng et al., 2011; Elhauge, 2010; Enthoven, 2009; Stange, 2009). Fragmentation involves multiple independent entities operating without synergy, resulting in duplicated services, and missed opportunities to meet patients’ comprehensive needs (Agha et al., 2017; Kern et al., 2019; Siqueira et al., 2021). Populations with long-term and complex care needs, such as children born with disabilities, are especially vulnerable to the negative effects of fragmented care. These individuals require continuous sociomedical care, an integrated approach that combines medical treatment with social support services addressing factors like medical, educational, and family counselling and assistance (Sipido et al., 2020). Discontinuity in treatment compromises care quality and adversely affects the quality of life and development of these patients (Cebul et al., 2008; Frandsen et al., 2015).

In Lebanon, the healthcare system is notably fragmented, with limited coordination between providers and scarce integrated sociomedical care. This fragmentation poses significant challenges for parents of children with disabilities, who often must navigate complex and disjointed care pathways. Historically, institutional care was the primary option for disabled children, but the recent shift toward home and community care has introduced new demands on families and healthcare structures. Despite the critical role parents play in managing their children’s care, most research on healthcare fragmentation has focused on providers and financers, overlooking parents’ lived experiences. This study aims to fill this gap by exploring the challenges faced by parents of disabled children within Lebanon’s fragmented healthcare system, highlighting their roles as informal care coordinators and “expert patients.”

## 2. Literature review: the lebanese healthcare system as a highly fragmented system

Prolonged decades of conflict brought with them a plethora of political, social, and economic challenges for Lebanon. Owing to the protracted wars and instability, the public sector was weakened, so the private sector took over as the main provider of services.

### 2.1. Lebanese healthcare governance

Owing to this precarious situation, public services were subpar, and governance ability was restricted (Khalife et al., 2017). With a combination of public and commercial payers and providers, the Lebanese healthcare system is now very fragmented (Ammar et al., 2007; Khalife et al., 2017).

This fragmentation has been further compounded by the increasing privatisation of healthcare services. The unchecked growth of the private sector, encouraged by lax governmental oversight, has led to the co modification of healthcare where profit motives often override patient-centred care (Ammar et al., 2007; Lerberghe et al., 2018). This privatised environment creates structural incentives for practices such as patient overbilling, supply-induced demand, and prioritisation of financially lucrative treatments, further entrenching fragmentation (Mossialos et al., 2005) and inequality in service provision. The decline of the public sector was matched by expansion and advancement in nongovernmental organisations (NGOs) and private institutions, which together account for the bulk of Lebanese hospitals (Khalife et al., 2017). Lebanese entrepreneurs have been drawn to participate in healthcare services as providers or financing agents because of their ability to form a business in a released atmosphere with low government oversight (Ammar et al., 2007). Healthcare professionals have been encouraged to commodify their services via the reimbursement system; the more hospital stays, surgeries, consultations, and diagnostic tests performed, the greater the income (Lerberghe et al., 2018). The culture of healthcare delivery in this privatised and fragmented system leads to what sociological theory describes as reification—the process by which social relations and human interactions become objectified and treated as things (Lukács, 1971; Honneth, 2008). In the Lebanese healthcare context, reification manifests in the depersonalisation of patients and the treatment of care as a commodity, contributing to behaviours such as concealing bad news and ignoring patient or parent perspectives. Lebanese healthcare service delivery prioritises profit maximisation over patient quality (Ammar et al., 2007).

### 2.2. Lebanese healthcare system organisation

The healthcare system in Lebanon is disintegrated in terms of professional provisions, funding, governance, and patient journeys. The Ministry of Public Health (MOPH) and the Ministry of Social Affairs (MOSA) are the two ministries that oversee the healthcare system. Medical services are governed by the MOPH. It is in charge of public hospitals and primary healthcare facilities. However, the organisation in charge of sociomedical services is the MOSA. Owing to its high degree of financial fragmentation across all financial aspects, the Lebanese healthcare system is highly fragmented (Siqueira et al., 2021). The MOPH, other social financing agencies, private insurance firms, and households are among the numerous financiers (WHO, 2017). Every financier has a different set of benefits, beneficiary groups, premium combinations, and a fixed copayment proportion. Owing to Lebanon's costly healthcare system and high out-of-pocket costs, homeowners are at risk for financial hardship. In Lebanon, the private sector is mostly responsible for providing healthcare services.

### 2.3. Patient medical pathway

Healthcare professionals are liberal professionals that are typically compensated on a fee-per-service basis and own their own private clinics. The coordination of patient care is non-existent. There is no gatekeeper on the patient journey. The choice of providers is left up to the patients. Table 1 below summarises the characteristics of the Lebanese healthcare system.

**Table 1.** Characteristics of the Lebanese healthcare system.

Feature	Characteristics
Access	Absence of gatekeeper Free circulation inside the system
Patient choice	Free choice of physicians Free choice of healthcare establishments (primary care, hospital, ambulatory…)
Benefits	Long term illness fully covered. Sociomedical services not fully covered and excluded by some financers. Dental and Glasses excluded
Financing	Copayment Private insurance to cover co-payment. Multiplicity of programs. Widespread informal payment
Renumeration of providers	Fee per service
State role	Limited power of the MOPH and MOSA

Prepared by the first author, inspired by (Kutzin, 2011).

The relationship between providers is informal and occasionally nonexistent, and there is no national regulatory structure in place for referrals. The same applies to the relationships among primary, secondary, and tertiary care. The patient starts treatment or seeks a physician of their choice on the basis of the advice of family members, friends, pharmacists, colleagues and regardless of his or her specialty.

**Figure 1.**
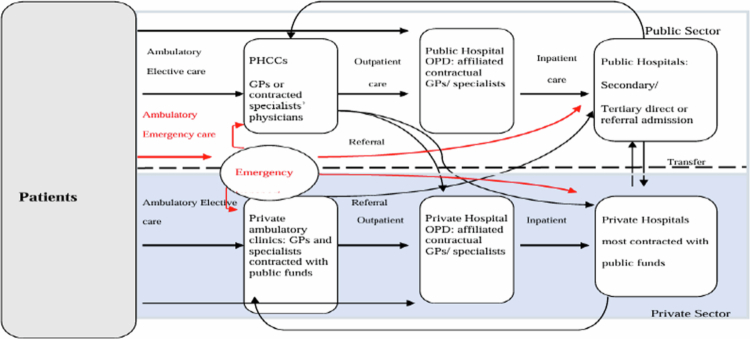
Patient medical pathway. Source: (Kosremelli Asmar & Yeretzian, 2019)***.***

The patient medical pathway presented in Figure 1 above gives an idea about the fragmentation of the Lebanese medical system where services provisions are mainly concentrated in the private sector section with the absence of a well-defined relation among various stakeholders.

With a mixture of public and private providers and financiers, the system is fragmented. The current referral system, which is restricted to administrative matters pertaining to hospital admittance at the expense of public funds through a visa centre issuing permission with the intention of controlling the bed supply, does not currently link primary care to secondary care (WHO, 2012). According to Lerberghe et al. (2018), the system is deficient in-patient routes, referral procedures, and coordination and/or continuity of care.

The Lebanese healthcare system serves as a perfect model of a highly fragmented healthcare system with private sector dominance. The effects of this fragmentation are particularly apparent in people with disabilities, who frequently require complicated healthcare treatments, including both medical and nonmedical settings, over an extended period of time (Murphy et al., 2011; Robben et al., 2012). This setting fits the purpose of our study highlighting the challenges faced by patients in highly fragmented healthcare systems.

## 3. Methods, data collection and analysis

### 3.1. Ethical considerations and study context

This study is a part of a doctoral research (Ghannam, 2022) that started in 2015 and was defended in 2022 at the Doctoral School of Economics and Business Sciences of the University of Nantes (École Doctorale Sciences Economiques et Sciences de gestion- EDGE). In terms of research committee ethical approvals, the French law obligation is limited to research in biomedical field. In France, the Code of public health (Article L. 1121-1) (resulting from the Jardé law) prohibits the start of research involving the human person (RIPH) without having first obtained a favourable opinion from the Committee for the Protection of Persons (CPP). This is mainly related to research in the biomedical field. For other research, there is no requirement to obtain ethical approval, although this is a good practice. The French decree dated 16 May 2016, (Ministère de l’Éducation nationale, de l’Enseignement supérieur et de la Recherche, 2016) setting the national framework for training and the procedures leading to awarding a doctoral degree in France did not require the prior ethic committee approval. It emphasises that the doctoral student should receive training in research ethics and scientific integrity. When we started our research, the EDGE did not require an ethics committee approval prior to conducting the research. However, the authors ensured the compliance with the code of ethics applied in research involving human participants.

### 3.2. Research design and methodological approach

Our main goal is to gain a thorough understanding of the difficulties faced by parents of children with disabilities in a healthcare system that is excessively fragmented. The research goal influences the methodological selection, which is not neutral (Charreire-Petit & Durieux, 2014:57–80). As a result, our method is exploratory; it is initially inductive before becoming abductive. The continuous backward and forward switching between the mobilising ideas and the observed data is a defining feature of the abductive technique. Taking into account the themes that surfaced from our initial literature review, we began our investigation. As a result, we were able to align our conceptual framework and methodology in the field. It also enabled us to pinpoint some initial research needs. By alternating among the field and the theory, we were able to improve and expand upon our conceptual categories. First, we conducted an exploratory qualitative study to learn about the difficulties patients face when navigating a highly fragmented healthcare system. Our attention might now be drawn to the participants’ actual experiences in their native environments. These are the best study techniques for learning about the interpretations and perspectives that participants assign to certain processes, structures, and events, including what they experienced and how they experienced it.

This made it possible for us to delve deeply into the issue and understand the phenomenon being studied from the standpoint of what it meant to each individual (Wacheux, 1996). This allowed us to recognise a phenomenon involving extremely fragmented healthcare systems. We then gathered information from the parents of those affected by this occurrence and created a synopsis of its main features. The questions of “what” the obstacles are and “how” the participants encountered them are addressed in this description (Creswell, 2007). The information above suggests that qualitative methodologies are necessary for our research. Our research is best suited for qualitative approaches since it provides a thorough grasp of the difficulties parents in a highly fragmented system confront. Individuals are given the opportunity to express their emotions and stories through qualitative research, which gives the participants’ voices a voice (Creswell, 2007).

### 3.3. Participant selection and sampling strategy

Since our goal is to research a phenomenon, we recruited participants via a purposive and selective sample technique to ensure that they could provide comprehensive and in-depth information regarding the phenomenon under study. As a result, we developed a set of standards for choosing participants. The participant’s familiarity with the topic being studied is the primary requirement. We searched for individuals who, while having similar experiences, differ in both their personal traits and their experiences. The requirements for inclusion were as follows: 1. The participant had to be the parent of a child born with a disability; 2. The disability had to be identified at birth or in early childhood; and 3. The individual with the disability had to be at least ten years old for the family to have enough experience raising a person with a disability. Given the diverse disabilities represented in our sample, including Down syndrome, autism, cerebral palsy, mobility-related disabilities, hearing and vision impairments, and developmental disorders, we recognise that parental experiences may vary significantly. This diversity enriches our data by capturing a broad spectrum of challenges but also requires careful interpretation of findings, acknowledging that certain experiences might be disability- specific. Ten participants had a combination of public funds, private insurance, and out-of-pocket expenses; seven participants had a mixture of private insurance and out-of-pocket expenses; three participants relied exclusively on out-of-pocket expenses; and two participants relied only on a public fund scheme. The participants’ coverage schemes varied. We contacted 45 families with members who were born disabled and who resided in Lebanon; 29 of the parents gave their consent to share their stories for the study’s purposes. We intended to include people with a variety of disabilities to gain a complete understanding of the experiences of the parents of children born with disabilities. The selected individuals were split as follows: seven volunteers who had a child with Down syndrome, four with autism, three with cerebral palsy, four with a disability related to mobility, one with hearing impairment, one with vision impairment, and eight with various developmental disorders.

### 3.4. Data collection procedures

Since the field of study is sensitive, we collected data according to conventional protocols (Gagnon et al., 2019). During the data collection and analysis process, we respected the protocols in order since a sensitive field research might have an impact on both the research and the participants. Semi structured interviews with parents of children with impairments were used to gather data. The interviews were conducted by the first author. The first author personally contacted each parent to arrange an interview according to their preferences. Each participant was contacted prior to the interview, the objective of the study was explained, and a meeting or call date had been set. At the beginning of the meeting, the first author obtained the participants informed oral consent to participate in the study, ensured to record the participants’ oral consent to participate in the study, to record the interview, and to publish the research. The majority of the interviews were conducted in person, but owing to COVID-19, we had to use online methods for some of them. The verbal consent was used as an alternative to written consent in remotely conducted interviews as the only available option especially during COVID-19 pandemic, it was also used in other interviews to foster a more open and less stressful participation in a this highly sensitive field of study. In semi structured interviews, participants are guided by the researcher’s precise questions while still feeling free to share their experiences. This is where most of our data come from. We conducted semi- structured interviews for all twenty-nine of them. The researcher’s extended guide served as the basis for the interviews, which were pretested by two exploratory interviews with parents of disabled children; the first parent had a child born with a mobility limitation, whereas the second parent had a developmental disability. We created an interview guide with open-ended questions based on these interviews to assist the participants in delving deeper into the subjects they find significant. The interview guide was broken down into three sections: the social background, the services needed, the funding information, and the sociodemographic data. This made it possible to fully comprehend the background and how it affected the parents’ experience.

### 3.5. Data analysis and coding strategy

To find important themes and patterns, we used thematic analysis (Saunders et al., 2015). Since the investigation was exploratory in nature, we began with an inductive open-coding methodology. This method frees the research findings from the structured methodologies and allows them to arise from the dominating or recurring themes present in the raw data (Thomas, 2006). Using Nvivo 11 software, we coded and then organised the codes into themes on the basis of verbatim merging. The coding followed an abductive approach; it was based on both the literature and data emerging from the field, which aligns with our aim to adopt an open, qualitative approach. This approach allowed themes and meanings to emerge organically from the participants' narratives, rather than being constrained by a rigid theoretical framework. It is particularly suited to exploratory research in under-studied contexts, such as the lived experiences of families of children with disabilities in Lebanon, where flexibility and sensitivity to participants’ unique social and cultural environments are essential.

We began with open, inductive coding to allow themes to emerge directly from the interview data. This was followed by an abductive approach, where we iteratively refined and organised the codes by relating them back to relevant literature and theoretical frameworks. Using NVivo 11, codes were continuously updated and grouped into broader categories to capture the complexity of participants’ experiences within the fragmented healthcare system.

Our coding strategy, which is a combined method based on both the literature and data emerging from the field, is consistent with our methodological approach. Our codes changed as the analysis progressed and were never static. Two categories made up our first order of codes: the first had twelve subcodes, and the second had five. Using an abductive method, we observed that the subcodes are dispersed across several layers. The issues fall into one of three categories: issues pertaining to the parent–provider relationship; issues pertaining to the patient care pathway; or issues pertaining to the system coverage structure. As a result, we have categorised the difficulties into these three topics under these issues as summed up in Table 2 below.

**Table 2.** Second-order Code.

Code level 1	Code level 2	Code level 3
Problems	Parent provider Interaction	Concealing the bad news
		Incomplete information and medical jargon
		Parents perceived as a goose with golden eggs
		Parent’s voice muted
	Patient care path	Lack of support units
		Informational discontinuity
		Geographical scattering of healthcare professionals
		Relational discontinuity
		Scarcity of some assistive devices and services
	System coverage structure	Bureaucracy burden
		Financial burden/Rigid ceiling
		Financial burden/Exclusion of services and assistive devices from the coverage

Source: First author’s compilation.

## 4. Results

### 4.1. The cost of fragmentation: financial strain and bureaucratic hurdles in Lebanon’s healthcare system

Two primary challenges to the healthcare system’s structure are revealed by the analysis of the interviews: the cost of providing services and the administrative complexity of approval procedures. The latter challenge revealed two primary subthemes: the exclusion of specific therapies and assistive devices and the ceiling of the coverage schemes. Parents have identified one of the main problems with the care and services that are provided to their children: the burden of the bureaucracy.

To receive partial reimbursement for the purchase of specific services and devices, parents must obtain preapproval. One person called the preapproval process a difficult experience.

Parents *“suffered”* from the bureaucracy of the approval processes and procedures.

*“You suffer a lot, lot of bureaucracy… You suffer a lot to get an approval.”* (Participant 9)

*“Don’t remind me of the suffering with the funding agency. The agency covered only 10 sessions per month and 15000 per session and the suffering, and bureaucracy…don’t remind me of those days....it has to be renewed monthly.”* (Participant 26)

Some participants explained that they stopped claiming reimbursement or coverage from public funds because of their complex bureaucratic procedures. As one parent explained,

*“Although I had public coverage, I never used it. It is a long and tiring process. I tried to use it once to cover a surgery, but I had to wait for a long time, so I gave up and never used it again.”* (Participant 5)

This bureaucracy amplified the financial burden of caring for a child with a disability. Providing long-term funding for various care services and possibly certain assistive devices is necessary while caring for a child with a disability.

One mother (Participant 10) said*, “Kids with disabilities need money.”* A parent declared that sometimes the scarcity of the “*financial means may stop you from reaching your goal.”* (Participant 22)

We attempted to pinpoint the primary causes of the financial hardship faced by parents of disabled children to gain a better understanding of it. Two primary subthemes stand out: the strict coverage cap and the exclusion of services and assistive devices from the programs covered. The maximum number of treatment sessions per patient per year is limited by the coverage plans, whether they are public or private. These therapies had to be paid out of pocket by the parents.

*“I had problems financing the physiotherapy sessions because the MOPH covers only two sessions of physiotherapy.”* (Participant 9)

*“The NSSF covered only 20 sessions of physiotherapy, then I started to pay out-of-pocket until I reached a phase when I was unable to pay for it at that hospital.”* (Participant 4)

The insurers imposed two different kinds of ceilings: one that was tied to the number and frequency of sessions over a given period of time and another that was tied to the annual or disease-specific monetary amount covered. The maximum number of sessions each period for the coverage plans was disclosed by the participants. Regardless of the requirements of the scenario, the ceiling is fixed:

Certain therapies, including speech and motor therapy, were not included in the coverage schemes' cap.

*“We have 100% coverage insurance, but they refused to cover my son’s treatment because he has a congenital disease.”* (Participant 27)

*“…My problem was not with the hospital admission fees; it was with the cost of the socio medical sessions.”* (Participant 6)

As several participants disclosed, parents occasionally had to end treatment sessions because of the inability to pay for financing.

*“My daughter has been having psychology sessions for 3 years; now I cannot afford the psychologist session fees, so we stopped the treatment.”* (Participant 18)

In Lebanon, financing agents do not reimburse the out-of-pocket costs associated with sociomedical services. For some families, out-of-pocket expenses present a significant challenge. As one mother revealed,

*“I am financially drained… I could not afford (the cost of treatment) anymore.”* (Participant 28)

In this study, we attempted to calculate parents’' out-of-pocket expenses starting at the child’s birth and continuing through the interview date. Families had to recollect details that they might have completely forgotten, which made the task challenging. As a result, we made an effort to aid in their memory of the frequency and session fee. The costs of education and sociomedical services were the two primary expenses that the participants could recall. The number of times that participants truly paid for doctor consultations was actually lost; as a result, we included only important interventions at the expense of medical services.

**Table 3.** Estimated out-of-pocket care payments: the case of a family with a child with Autism.

Case	Disability	Diagnosis	Date of birth	Cost till the day of the interview in USD	Cost per year per USD
10	Autism	Early childhood	2006	540,193	8,585
19	Autism	Early Childhood	2002	254,213	14,123
23	Autism	Early Childhood	2003	533,173	31,363
28	Autism	Early Childhood	2003	330,320	19,431

Source: Prepared by the first author.

**Table 4.** The challenges according to their density in descending order.

Category	Code	Frequency of codes	Recurrence of codes
System coverage structure	Financial burden: ceiling of coverage schemes and exclusion of services and devices	28	94
Patient care path	Need for support units	15	34
Parent- Provider interaction	Incomplete information and medical jargon	14	33
Patient care path	Informational discontinuity	11	27
Parent- Provider interaction	Parents ‘voice muted	10	20
Patient care path	Geographical scattering of service providers	10	21
Parent- Provider interaction	Concealing the bad news	11	19
Patient care path	Relational discontinuity	11	19
Parent- Provider interaction	Parents as a goose with golden eggs	9	15
Patient care path	Scarcity of certain assistive devices or services	6	11
Parent- Provider interaction	Not false hope but encouragement	7	9
System coverage structure	Bureaucracy burden	6	6

Source: Prepared by the author.

Table 3 above shows that the annual cost of caring for a child with autism ranges from $14,123 to $38,585, which is substantial in the Lebanese context. To provide perspective, the average annual salary in Lebanon is estimated to be approximately $7,500 (Lebanese Central Administration of Statistics, 2023). Additionally, at the time of data collection, the minimum wage in Lebanon averaged around $500 per month, or about $6,000 annually, underscoring the limited financial capacity of many families. This means that for many households, the costs of autism care far exceed typical earnings, representing a significant financial burden. These costs do not include medical expenses, which are typically covered through a mix of public and private funding sources.

The majority of parents interviewed are university graduates, which aligns with the higher education rate in Lebanon which was 56% for female 25 + years in 2018 and gross rate of population 25 + years was 33% (CEIC, 2025). This indicates that the sample largely reflects families with a high level of educational background. Due to the demands of caring for a child with disabilities, some mothers were obliged to quit their jobs as they could not manage both caregiving and employment. However, some mothers later returned to work once their child’s condition allowed. While some families reside in rural areas, Lebanon’s small geographic size means that living in a village does not necessarily limit access to services; most families can reach urban centres within a 1 to 2-hour drive, ensuring access to available autism-related services.

According to our analysis, the healthcare systems’ complicated and dispersed structure is mostly to blame for the burden. The financial load is the most significant factor. Ninety-four times the financial strain is mentioned by twenty-eight out of the twenty-nine participants as a serious difficulty. The patient and/or his family are not supported by the design of the healthcare system. The second major problem that the participants highlighted was this lack of assistance. Because there is no gatekeeper or case manager at the entry point in the Lebanese healthcare system, parents are forced to take on the roles of patient expert and case manager. The contact between the patient and the provider gives rise to the third key issue. Insufficient information on their children’s cases is given to the parents. The recurrence of codes identified by the analysis of the empirical data is shown in Table 4 below, arranged in decreasing order. The interviewees specifically stated or defined these codes as presenting issues. The frequency of the code refers to the number of participants who mentioned this code. The recurrence of the code is the number of times this code was mentioned in all the interviews.

**Table 5.** Roles played by parents and their responsibilities.

Parents’ role	Responsibilities
Patient expert	‐ Has Knowledge about condition and treatment condition. ‐ Has self-management skills.
Case/Care path manager	‐ Chooses the treatment. ‐ Coordinates between providers. ‐ Negotiates with the financers

Source: Prepared by the first author.

### 4.2. Reification and relational challenges: parents’ struggle for recognition in disability care

The difficulties parents of disabled children encounter as a result of the fragmentation of the Lebanese healthcare system were made clear by our data analysis. The relationships between the parent and the provider, the care path, and the structure of the Lebanese healthcare system were the three key levels at which these challenges were found. We explain in detail that the fragmentation of the healthcare system led to the reification of parents and their child born with a disability.

The initial exchange between the parents and the caregivers is at the time of the diagnosis of the child’s case. The effectiveness of these interactions affects parents’ contentment or discontent with the calibre of care they receive, as well as the care path and care outcome (Keen, 2007). Our analysis allowed us to identify five recurrent key points characterising the comportment of the healthcare provider as dehumanising the relationship between caregivers and parents:

‐ The bad news was hidden and not announced.
‐ The information provided was vague and provided via medical jargon.
‐ The healthcare team members did not encourage the parents or give them hope.
‐ The parents thought that they were considered like a goose with golden eggs.
‐ The parents felt muted, which means that the healthcare providers did not listen to them.

The parents interviewed stated that they were not notified of their child’s condition by medical experts. As a result, parents believe that medical staff do not assume responsibility for disclosing their child’s medical information. The majority of the participants learned of their children’s disabilities by accident. For example, a mother accidentally conversed with two other nurses.

*“While I was entering the newborn yard to breastfeed my son, I heard one nurse telling her colleague that my son is down syndrome.”* (Participant 6)

While some participants communicated their displeasure to the care provider, others saw this attitude as an attempt to withhold information from them. When a mother discovered by accident that her child had Down syndrome, she addressed the medical staff.

*“You do not have the right to hold such information from me; I am her mother, and I need to know. If my daughter is trismic, it is not your fault, and I need to know it.”* (Participant 11)

The parents believed that the healthcare personnel were withholding information concerning their child’s impairment or the course of therapy. “*You need to beg the physician to tell* (the parents) *what to expect*,” Participant 16 said. Information exchange is regarded by parents as a fundamental necessity when they manage their child’s condition. The parents frequently do not understand the medical jargon or technical phrases that the doctors use while they share information. Participant 1 said that she received a medical report upon the newborn’s release but that she was unable to comprehend any of it and that “*the doctors did not explain*” it.

In the context of disability, a communicative paradox arises from the conflict between hope and reality. When parents receive unexpected news about a disability, they rely heavily on support and encouragement from others to help them deal with the situation. Parents believe that healthcare providers have a duty to be supportive. Nonetheless, a few individuals conveyed their discomfort with the “negative remarks” made by the provider.

*“We had lots of negative expectations from the experts: “Do not imagine that he could walk one day, that he would play one day….” Do not put hope so high. At this stage, faced with all the negative feedback, we decided that we need to educate ourselves on the case.”* (Participant 2)

Participants 1 and 9 almost *“collapsed* “when the physicians announced in a factual manner that their sons might die soon without making sure first that the parents were aware of the gravity of their children’s disease and without preparing the parents for such drastic news.

Parents feel that they are funding healthcare providers in a fragmented, profit-driven system where healthcare coverage is minimal and out-of-pocket costs are high. The parents were truly dissatisfied with the way the experts acted. They see them “s *"materialistic* “and “*greedy*” (Participant 7). Instead of billing patients for individual services, healthcare practitioners were paid on an appointment basis. There is no strategy for financing long-term treatment. “*Some professionals made a fortune. we (parents of a child with disability) are a goose with golden eggs*,” Participant 23 mentioned.

Professionals also fail to value parents’' perspectives, according to them. It was as if their voices were being silenced. To comprehend their emotions as parents, they desired that the experts delve deeper than the clinical components (Gómez-Zúñiga et al., 2019). They think that they know their kids better than anyone else does. While medical experts were fixated on the illness, parents were able to take a more holistic view of their child, as they learned more about his or her strengths, weaknesses, and preferences every day. The parents’ expertise was ignored by professionals, who rarely listened to them or took their opinions into account. Parents “*wanted to be partners*” in the healthcare team’s efforts to treat their children. Involvement in care decision-making by providers is what they desire. Recalling a discussion with an ergo therapist, one mother said:

*“…she is my daughter, you (the therapist) will sit or stay with her quarter an hour, but I am living with her a whole life, and you need to take my opinion into consideration. You cannot make decisions by yourself!”* (Participant 8)

Because of this, we must once again consider the patient’s or family’s position inside the healthcare system and consider the new trend of expert patients. This new trend includes patients, their families, or representatives from their families as integral parts of the healthcare team. According to Boulet (2016), “expert patient is a patient who has a significant knowledge of his/her disease and treatment in addition to self-management skills”. Skilled patients are able to express themselves clearly to their doctors, offer constructive criticism of treatment plans, and participate in decision-making processes. Even more so, they can have a hand in writing healthcare standards (Cordier, 2014). As a result, medical schools should establish patient education programs and devote specialised training to the value of the “expert patient” in healthcare systems (Friconneau et al., 2020), training that already exists in some countries.

Furthermore, the elements characterising the comportment of healthcare professionals lead us to conclude that the situation lived and experienced by parents could be described as reifying by the users of healthcare systems, parents and children.

Axel Honneth (2007) theorised the notion of reification by resuming Georg Lukacs’ work, who utilised this term to characterise the human relationship in a context criticising capitalism.

According to Honneth, the reification resides in negating the recognition of others. He identifies two models in which reification might take place.

The first is the mechanised exchange context. When healthcare providers announce their diagnosis and do not take the parent point of view into consideration, they even ignore it, which conforms with the reification first model.

The second model is related to an overlooking ideology that influences the conduct of an individual to the extent that he or she forgets to recognise what preexists.

When a physician renounces the expertise of the patient, refuses to include him or her in the healthcare team and seems to give priority to the monetary perspective of the relationship, he or she becomes reifying and does not recognise the patient who is limited to a detained payer.

### 4.3. From caregivers to case managers: parental roles in navigating a fragmented healthcare system

Caring for people with disabilities necessitates the participation of numerous health professionals over a prolonged period of time. Owing to their constant presence from birth, parents have the power to greatly impact their disabled child’s health (Resch et al., 2010). Resch et al. (2010) reported that parents may face challenges while navigating a fragmented healthcare system, which can impact both the quality of care their child receives and their overall wellness. Our research revealed five overarching themes concerning the difficulties encountered by parents navigating the care pathway for children with disabilities. The first issue is the dearth of support groups; the second is the informational gap; the third is the dispersion of healthcare providers across the country; the fourth is the break in interpersonal relationships; and the fifth is the dearth of specific assistive technology and services. Upon learning of their child’s condition, the parents did not receive any psychological help from the Lebanese healthcare system. When the news of the impairment was announced, the parents were left all alone. Most of them vividly recall what happened and characterise it as a catastrophe.

Express his feelings, a father said:

*“It was the Day (c’ était le jour J de l’ enfer qui a déclenché tout) of hell that triggered everything.”* (Participant 21)

Another parent summed it all “.

*"Most of the parents, when they get a child with a disability, feel that the world stopped.”* (Participant 26)

Informing their parents of their children’s impairment left them feeling bewildered. They were not escorted along the care pathway by healthcare providers. There was a lack of assistance along the care pathway following the disclosure of a new illness or condition. Although cardiac conditions are prevalent in people with Down syndrome, one mother reported feeling as although she “*died for the second time*” upon learning of her daughter’s diagnosis from a cardiologist.“A “*treasure-seeking journey*” was the analogy that another mother used to describe the time her child spent in speech therapy.

The informational gap is another major challenge. People with disabilities may suffer from more than one medical condition, necessitating the care of a wide range of specialists. It is common practice to move people with disabilities between different types of healthcare facilities, such as primary and secondary care facilities. The experts do not manage the patient’s transition between care units in Lebanon’s extremely disintegrated healthcare system. They provide services within the context of their clinic and their area of expertise.

As a result, there is an absence of coordination among healthcare service providers even though they might be working on the same case simultaneously. To facilitate the transfer and transmission of necessary information to the next provider, the majority of participants did not even obtain a written medical summary of their children’s illnesses. According to one parent, their child was prescribed physical therapy by an orthopaedist, but the doctor neglected to give the physiotherapist any kind of report outlining the child’s illness or providing any background information.

*“I have never got a report, or a letter addressed to the physiotherapist or on what to focus during the treatment session.”* (Participant 3)

Parents, particularly those without medical experience, felt “*lots of stress*” because they were supposed to coordinate with many doctors. They saw themselves as “*the link*” connecting the providers.

*“I was the link between the physiotherapist and the doctor. The doctor wrote a physiotherapy demand addressed to the insurance company to obtain coverage approval. I presented my son's case to the physiotherapist; I explained what was expected from these sessions.”* (Participant 1)

On the other hand, there is no central database for patients in Lebanon's healthcare system.

*“There is no complete file; each physician has part of my son’s medical file.”* (Participant 25)

The participants were faced with the daunting task of gathering information about their child’s problems. One mom voiced their dissatisfaction, saying, “*nobody has a file of (her) son's case only me!!*” (The12th participant). Some parents requested that their child’s doctors write up a summary of their child’s diagnosis and treatment plan so that they could easily explain their child’s situation to a different doctor if necessary. The delivery of healthcare in Lebanon, like in other countries with fragmented systems, is typically dispersed across many locations, as participant 10 complained that *“there is no multidisciplinary team. Thus, he (her child) cannot have all the treatments in one place.”*

For families already struggling to meet their children’s basic requirements, this adds even more stress. The dispersed locations of the care clinics caused concern among the parents. They had to commute to the other clinics. “*Spending a life running*” to make up for missed treatment appointments was one parent’s gripe.

*“I used to run from one place to another to be able to attend a session, until now. We spent our life running, running.”* (Participant 25)

Sometimes parents sacrifice their own sleep, money, and time with other children in pursuit of therapies. “*Physically and financially*,” the parents were worn out. Some parents were forced to leave their jobs; one mother shared that she had to decide between her career and taking her child to treatment appointments, so she left her work.

*“I used to be a teacher, but I quit when I got my son because I had to go from one therapist to another, so it became difficult, and I could not make it all.”* (Participant 28)

Liberals who work in healthcare often work for private practices and clinics. The parents had to seek out experts whenever treatment was necessary. They “*shopped*” about to get the “*right healthcare professionals*.” The mother shared that she sought out “a “*good therapist*” after working with more than 10 different speech therapists.

*“I started to look for a speech therapist. I cannot tell you how many therapists I visited… I had changed more than 7 or 10 therapists over a short period of time. This is a waste of time for my son.... We changed many speech therapists until we met her, she was good, helpful, and my son liked her.”* (Participant 17)

One of the difficulties parents with disabled children encounter is the scarcity of appropriate assistive technology. To better assist their disabled children, some participants sought out information on their own. The parents set out to discover tools that would aid in their children’s growth and development. Take the case of a toddler who can neither speak nor move. The eye tracker was one of several communication devices that his father saw online when looking for a device for his child. He imported one so that his son could use eye movements to type. The eye tracker allowed the child to communicate and permitted him to join school.

*“My husband is a computer engineer, so he researched all the time how technology could help us. We need to create a means of communication with our son. He found a tool called the board; the person could use his eyes to type.”* (Participant 12)

These findings highlight two new responsibilities for parents in addressing the shortcomings of the healthcare system, which is very fragmented. In the preceding part, we established the patient expert as the primary figure. The second role the parents play is directing their children’s care and becoming the case or care path manager. A case or care path management function is being responsible for selecting the best course of treatment, coordinating patients' care, and making the most efficient use of available resources (Fabbri et al., 2017). The roles of the parents are summarised in Table 5.

The participants were treated as integral parts of the healthcare team because of their expertise as patients. The parents’' responsibilities as care path managers included making provider and treatment plan selections, facilitating communication between healthcare providers, and guaranteeing adequate funding for services. The identification of holes in the extremely fragmented healthcare system led to the emergence of these parental roles.

## 5. Discussion

The Lebanese healthcare system is dominated by the private sector (Ammar et al., 2007; Lerberghe et al., 2018). This dominance accompanied with limited governmental oversight intensified the systemic implications of the privatisation of the healthcare system notably the health inequity and discontinuity of care. This finding is consistent with the results of research on the limitations of the privatisation of the healthcare systems (Borsa et al., 2023; Goodair & Reeves, 2024).

This study also highlights the profound impact of a fragmented healthcare system on parents of disabled children in Lebanon. The complex bureaucracy and strict coverage ceilings contribute heavily to financial strain, which was the predominant challenge reported by participants. This finding aligns with prior research showing that out-of-pocket costs pose significant barriers in fragmented healthcare systems.

The absence of a gatekeeper or case manager forces parents to assume multiple roles—not only as caregivers but also as care coordinators and advocates. This added responsibility creates an overwhelming burden, compounded by poor communication and informational discontinuity between providers, consistent with findings from other fragmented healthcare settings (Brewer, 2018).

Moreover, the dehumanising interactions experienced by parents, such as withholding bad news, the use of inaccessible medical language, and failure to acknowledge parents’ expertise, reflect a systemic issue of “reification,” where patients and families are reduced to mere objects within the healthcare system (Honneth, 2007). Recognising parents as “expert patients” with valuable knowledge and involving them in care decisions could improve both the quality of care and family satisfaction (Boulet, 2016).

The scarcity of assistive technologies and the scattered nature of services further exacerbate the logistical, emotional, and financial challenges faced by families. These findings underscore the urgent need for integrated care models that offer coordinated, multidisciplinary services in accessible locations, alongside psychosocial support starting at diagnosis.

## 6. Implications

To improve the current state of care for families of children with disabilities, several policy and systemic changes are necessary. There is a need for regulatory safeguards that limit the drawbacks of the privatisation of the healthcare system; regulations that ensure the access to care for vulnerable groups and limit profit-driven practices to improve the equity and continuity of care. The coverage schemes should be revised to eliminate rigid caps on services and simplify reimbursement bureaucracy, reducing the financial burden on families. Implementing formal case management roles would support families in navigating the healthcare system and coordinating care. Healthcare professionals must receive training aimed at improving communication and fostering partnership with families. Promoting the “expert patient” model can empower parents and enhance care outcomes. Additionally, medical education institutions should restructure curricula to emphasise family-centred care, communication skills, and interdisciplinary collaboration. This will prepare future healthcare providers to meet the complex needs of children with disabilities and their families more effectively. Further research should extend this work to other Middle Eastern contexts and evaluate interventions designed to reduce healthcare fragmentation and better support affected families.

## 7. Conclusion

This study has discussed the difficulties faced by parents of disabled children due to a fragmented healthcare system. We have taken an objective look at how the fragmented system has failed to meet the requirements of families dealing with disabled children. Since there was a lack of coordination among the care providers and a discontinuity in the flow of information and relationships throughout the treatment path, our study revealed that the majority of participants took on the dual roles of patient experts and case managers. This work calls for similar research in other highly fragmented healthcare systems in the Middle East as well as in the Occident, especially in contexts where governments are making more room for the private sector.

## Ethics declaration

In France, the Code of public health (Article L. 1121-1) (resulting from the Jardé law) prohibits the start of research involving the human person (RIPH) without having first obtained the favourable opinion of a Committee for the Protection of Persons (CPP). This is mainly related to research in the biomedical field. For all other research, there is no requirement to obtain ethical approval. This information was obtained after contacting « The Haut Conseil de l'évaluation de la recherche et de l'enseignement supérieur (Hcéres) » .

The authors declare that the research meets the ethical guidelines of the country of the study. Nantes University doctoral business school did not require an ethic committee approval prior to conducting the research. However, doctoral students were entitled to receive training in research ethics and scientific integrity. The first writer, receiving her doctoral diploma in 2022, fulfilled the requirement law dated 25 May 2016 and swore on respecting the research code of ethic and the scientific integrity.

## Consent declaration

The participants gave an oral consent that has been recorded at the beginning of each interview. Data collection was obtained through interviews. The interviews were conducted by the first author. Each participant was contacted prior to the interview, the objective of the study was explained, and a meeting date had been set. At the beginning of the meeting, the first author recorded the participants’ oral consent to participate in the study, to record the interview, and to publish the research.

## Data Availability

The data supporting the findings are available upon request from the corresponding authors upon reasonable request.

## References

[cit0001] Agha, L., Frandsen, B., & Rebitzer, J. B. (2017). Causes and consequences of fragmented care delivery: Theory, evidence, and public policy. *National Bureau Of Economic Research*, *50*. https://www.nber.org/system/files/working_papers/w23078/revisions/w23078.rev1.pdf

[cit0002] Ammar, W., Wakim, R., & Hajj, I. (2007). Accreditation of hospitals in Lebanon: A challenging experience. *Eastern Mediterranean Health Journal*, *13*, 138–149. https://www.researchgate.net/publication/6289493_Accreditation_of_hospitals_in_Lebanon_A_challenging_experience 17546916

[cit0003] Borsa, A., Bejarano, G., Ellen, M., & Bruch, J. D. (2023). Evaluating trends in private equity ownership and impacts on health outcomes, costs, and quality: Systematic review. *BMJ (Clinical Research ed.)*, *382*, e075244. 10.1136/bmj-2023-075244 PMC10354830 37468157

[cit0004] Boulet, L.-P. (2016). The expert patient and chronic respiratory diseases. *Canadian Respiratory Journal*, *2016*, 9454506. 10.1155/2016/9454506 27445572 PMC4904534

[cit0005] Brewer, A. (2018). We were on our own”: Mothers’ experiences navigating the fragmented system of professional care for autism. *Social Science & Medicine*, *215*, 61–68. 10.1016/j.socscimed.2018.08.039 30212758

[cit0006] Cebul, R. D., Rebitzer, J. B., Taylor, L. J., & Votruba, M. E. (2008). Organizational fragmentation and care quality in the U.S. healthcare system. *Journal of Economic Perspectives*, *22*(4), 93–113. 10.1257/jep.22.4.93 19791306

[cit0007] CEIC. (2025). Lebanon: Social – Education Statistics. Retrieved April 5, 2025, from https://www.ceicdata.com/en/lebanon/social-education-statistics

[cit0008] Charreire-Petit, S., & Durieux, F. (2014). Chapitre 3. Explorer et tester : les deux voies de la recherche, *Méthodes De Recherche En Management. Vol. 4e éd., Management Sup* (pp. 76–104). Paris: Dunod. 10.3917/dunod.thiet.2014.01.0076

[cit0009] Cheng, S.-H., Hou, Y.-F., & Chen, C.-C. (2011). Does continuity of care matter in a health care system that lacks referral arrangements? *Health Policy and Planning*, *26*(2), 157–162. 10.1093/heapol/czq035 20699348

[cit0010] Cordier, J.-F. (2014). The expert patient: Toward a novel definition. *European Respiratory Journal*, *44*(4), 853–857. 10.1183/09031936.00027414 25271227

[cit0011] Creswell, J. W. (2007). *Qualitative Inquiry and Research Design: Choosing Among Five Approaches* (2nd ed.). SAGE Publications.

[cit0012] Elhauge, E. (2010). *The Fragmentation of U.S. Health Care*. Oxford University Press.

[cit0013] Enthoven, A. (2009). Integrated delivery systems: The cure for fragmentation. *The American Journal Of Managed Care*, *15*(10 Suppl), S284–90. 20088632

[cit0014] Fabbri, E., De Maria, M., & Bertolaccini, L. (2017). Case management: An up-to-date review of literature and a proposal of a county utilization. *Annals of Translational Medicine*, *5*(20), 396–396. 10.21037/atm.2017.07.26 29152496 PMC5673790

[cit0015] Frandsen, B. R., Joynt, K. E., Rebitzer, J. B., & Jha, A. K. (2015). Care fragmentation, quality, and costs among chronically ill patients. *The American Journal of Managed Care*, *21*(5), 355-–62. https://pubmed.ncbi.nlm.nih.gov/26167702/26167702

[cit0016] Friconneau, M., Archer, A., Malaterre, J., Salama, F., & Ouillade, M.-C. (2020). Le patient-expert - Un nouvel acteur clé du système de santé. *médecine/sciences*, *36*, 62–64. 10.1051/medsci/2020206 33427642

[cit0017] Gagnon, M., Beaudry, C., & Deschenaux, F. (2019). « Prendre soin » des participants lors d’entretiens réalisés en contexte de recherches sensibles. *Recherches Qualitatives*, *38*(2), 71–92. 10.7202/1064931ar

[cit0018] Ghannam, D. (2022). *Resilience, Absorptive Capacity, and Compassion: The Case of the Parents of Children with Disability in a Highly Fragmented Healthcare System*. France: University of Nantes.

[cit0019] Gómez-Zúñiga, B., Pulido, M., Pousada, F., García, O., & Armayones, R. (2019). The experience of parents of children with rare diseases when communicating with healthcare professionals: Toward an integrative theory of trust. *Orphanet Journal of Rare Diseases*, *14*(1), 159. 10.1186/s13023-019-1134-1 31253163 PMC6599337

[cit0020] Goodair, B., & Reeves, A. (2024). The effect of health-care privatisation on the quality of care. *The Lancet. Public Health*, *9*(3), e199–e206. 10.1016/S2468-2667(24)00003-3 38429019

[cit0021] Honneth, A. (2007). La réification : petit traité de théorie critique, Gallimard, Essais.

[cit0022] Honneth, A. (2008). Reification and Recognition: A New Look at an Old Idea, *Reification and Recognition: A New Look at an Old Idea* (Vol. n.a., pp. 16–75). Oxford : Oxford University Press. 10.1093/acprof:oso/9780195320466.003.0002

[cit0023] Keen, D. (2007). Parents, families, and partnerships: Issues and considerations. *International Journal of Disability, Development and Education*, *54*(3), 339–349. 10.1080/10349120701488855

[cit0024] Kern, L. M., Safford, M. M., Slavin, M. J., Makovkina, E., Fudl, A., Carrillo, J., & Abramson, E. L. (2019). Patients’ and providers’ views on causes and consequences of healthcare fragmentation in the ambulatory setting: A qualitative study. *Journal of General Internal Medicine*, *34*(6), 899–907. 10.1007/s11606-019-04859-1 30783883 PMC6544669

[cit0025] Khalife, J., Rafeh, N., Makouk, J., El-Jardali, F., Ekman, B., Kronfol, N., Hamadeh, G., & Ammar, W. (2017). Hospital contracting reforms: The lebanese ministry of public health experience. *Health Systems & Reform*, *3*(1), 34–41. 10.1080/23288604.2016.1272979 31514709

[cit0026] Kosremelli Asmar, M., & Yeretzian, J. S. (2019). Lebanon: Health system review. Health Systems in Transition-Institute of Public Health- Saint Joseph University-Lebanon.

[cit0027] Kutzin, J. (2011). Bismarck vs. Beveridge: Is There Increasing Convergence between Health Financing Systems 1st annual meeting of SBO network on health expenditure, 21–22 November 2011, Paris, OECD. https://www.scribd.com/document/296077278/Bismarck-vs-Beveridge

[cit0028] Lerberghe, W. V., Abdelhay, M., & Nabil, K. (2018). *The Collaborative Governance of Lebanon’s Health Sector Twenty Years Of Efforts to Transform Health System Performance*. Ministry of Public Health- Lebanon.

[cit0029] Lukács, G (1971). History and Class Consciousness: Studies in Marxist Dialectics. (R. Livingstone, Trans.). Cambridge, MA: MIT Press.

[cit0030] Ministère de l’Éducation nationale, de l’Enseignement supérieur et de la Recherche. (2016). Arrêté Du 25 Mai 2016 Fixant Le Cadre National de La Formation et Les Modalités Conduisant à La Délivrance Du Diplôme National de Doctorat.

[cit0031] Mossialos, E., Allin, S., & Davaki, K. (2005). Analyzing the greek health system: A tale of fragmentation and inertia. *Health Economics*, *14*(Suppl 1), S151–168. 10.1002/hec.1033 16161195

[cit0032] Murphy, N. A., Carbone, P. S., & Council on Children with Disabilities. (2011). Parent-provider-community partnerships: Optimizing outcomes for children with disabilities. *Pediatrics*, *128*(4), 795–802. 10.1542/peds.2011-1467 21949138

[cit0033] Resch, J., Aaron, G., Mireles, M. R., Benz, C., Grenwelge, R., Peterson, D., & Zhang (2010). Giving parents a voice: A qualitative study of the challenges experienced by parents of children with disabilities. *Rehabilitation Psychology*, *55*(2), 139–150. 10.1037/a0019473 20496968

[cit0034] Robben, S., Huisjes, M., van Achterberg, T., Zuidema, S., Rikkert, M., Schers, H., Heinen, M., & Melis, R. (2012). Filling the gaps in a fragmented health care system: Development of the health and welfare information portal (ZWIP). *JMIR Research Protocols*, *1*, e10. 10.2196/resprot.1945 23611877 PMC3626145

[cit0035] Saunders, M. N. K., Lewis, P., & Thornhill, A. (2015). *Research Methods for Business Students*. Pearson Education Limited.

[cit0036] Sipido, K. R., Antoñanzas, F., Celis, J., Degos, L., Frackowiak, R., Fuster, V., Ganten, D., Gay, S., Hofstraat, H., Holgate, S. T., Krestin, G., Manns, M., Meunier, F., Oertel, W., Palkonen, S., Pavalkis, D., Rübsamen-Schaeff, H., Smith, U., Stallknecht, B. M., & Zima, T. (2020). Overcoming fragmentation of health research in Europe: Lessons from COVID-19. *The Lancet*, *395*(10242), 1970–1971. 10.1016/S0140-6736(20)31411-2 32559417

[cit0037] Siqueira, M., Coube, M., Millett, C., Rocha, R., & Hone, T. (2021). The impacts of health systems financing fragmentation in low- and middle-income countries: A systematic review protocol. *Systematic Reviews*, *10*(1), 164. 10.1186/s13643-021-01714-5 34078460 PMC8170990

[cit0038] Stange, K. C. (2009). The problem of fragmentation and the need for integrative solutions. *The Annals of Family Medicine*, *7*(2), 100–103. 10.1370/afm.971 19273863 PMC2653966

[cit0039] Thomas, D. R. (2006). A General Inductive Approach for Analyzing Qualitative Evaluation Data. *American Journal of Evaluation*, *27*(2), 237–246. 10.1177/1098214005283748

[cit0040] Wacheux, F. (1996). *Méthodes qualitatives et recherche en gestion* (Vol. n.a., pp. 290). Paris : Economica. Post-Print hal-00157140, HAL.

[cit0041] WHO (2017). *Primary health care systems (PRIMASYS): Comprehensive case study from Lebanon*. Geneva: World Health Organization. http://data.infopro.com.lb/file/Primary%20Healthcare%20Center%20WHO%202017.pdf

[cit0042] World Health Organization. Regional Office for the Eastern Mediterranean. (2012). Health System Profile-Lebanon. https://applications.emro.who.int/dsaf/EMROPUB_2014_EN_1745.pdf

